# ArthroRad trial: randomized multicenter single-blinded trial on the effect of low-dose radiotherapy for painful osteoarthritis—final results after 12-month follow-up

**DOI:** 10.1007/s00066-023-02152-z

**Published:** 2023-10-10

**Authors:** Marcus Niewald, Sobhan Moumeniahangar, Lara N. Müller, Matthias G. Hautmann, Yvonne Dzierma, Jochen Fleckenstein, Stefan Gräber, Christian Rübe, Markus Hecht, Patrick Melchior

**Affiliations:** 1 Department of Radiotherapy and Radiooncology, Saarland University Medical Center Homburg/Saar, Homburg/Saar, Germany; 2 Department of Radiotherapy and Radiooncology, Clinics of South-East Bavaria, Traunstein, Germany; 3 Institute for Medical Biometry, Epidemiology and Medical Informatics, Saarland University Medical Center, Homburg/Saar, Germany; 4 Mühlstraße 28, 66894 Bechhofen, Germany

**Keywords:** Osteoarthritis, Low-dose radiotherapy, Pain, KOOS-PS, SF-SACRAH

## Abstract

**Objective:**

Updated report about the randomized comparison of the effect of radiotherapy on painful osteoarthritis (OA) applying a standard dose vs. a very low dose regime after a follow-up of 1 year.

**Patients and methods:**

Patients presenting with OA of the hand/finger and knee joints were included. After randomization (every joint region was randomized separately) the following protocols were applied: (a) standard arm: total dose 3.0 Gy, single fractions of 0.5 Gy twice a week; (b) experimental arm: total dose 0.3 Gy, single fractions of 0.05 Gy twice a week. The dosage was blinded for the patients. For evaluation the scores after 1‑year visual analog scale (VAS), Knee Injury and Osteoarthritis Outcome Score–Short Form (KOOS-PS), Short Form Score for the Assessment and Quantification of Chronic Rheumatic Affections of the Hands (SF-SACRAH) and 12-item Short-Form Health Survey (SF-12) were used (for further details: see [1]).

**Results:**

The standard dose was applied to 77 hands and 33 knees, the experimental dose was given to 81 hands and 30 knees. After 12 months, the data of 128 hands and 45 knees were available for evaluation. Even after this long time, we observed a favorable response of pain to radiotherapy in both trial arms; however, there were no reasonable statistically significant differences between both arms concerning pain, functional, and quality of life scores. Side effects did not occur. The only prognostic factor was the pain level before radiotherapy.

**Conclusions:**

We found a favorable pain relief and a limited response in the functional and quality of life scores in both treatment arms. The possible effect of low doses such as 0.3 Gy on pain is widely unknown.

## Background

Osteoarthritis (OA) is a very frequent disease especially in elderly people. Caused by overweight, improper load of the joint, injuries, dysplasia, arthritis or other arthropathies, a progressive destruction of the joint cartilage may potentially involve the bone, the joint capsule and the adjacent muscles [2, 3]. Very frequently, OA causes pain. In the beginning, only repeated movements or burden applied to the joint are painful. Later, pain may occur during rest or at night. The joints are deformed; passive or active mobility are impaired.

The analgesic effect of radiotherapy on patients with OA has been known for a long time. There is a large body of retrospective publications showing a good analgesic effect of radiotherapy for osteoarthritis of the knee joint in 58–91% of patients [2], whereas literature reports on hand and finger joints are rare [4–6].

There have been research activities on arthritis models in order to clarify the mechanism of the effect of radiotherapy in OA treatment, which have led to an improved understanding. Radiation has been shown to inhibit the adhesion of macrophages to the endothelium, induces the expression of the x‑linked apoptosis inhibitor, of the transforming growth factor beta (TGFβ), reduces the expression of E‑ and L‑selectin and inhibits the expression of interleukin 1 (IL‑1) and the chemokine ligand 20 (CCL 20). All these effects are maximal after single doses of 0.3–0.7 Gy [7–9]. More recent research shows a shift in blood cell counts, while the total number of leukocytes remains unchanged and a general reduction in inflammatory cytokines [10, 11]. Furthermore, low-dose radiotherapy inactivates mitochondrial function [12]. The most recent overview of biological findings and their consequences for the clinical radiotherapy application has been given by Weissmann et al. [13].

We thus conducted a prospective randomized trial in order to examine the effect of radiotherapy on painful OA and to provide a high level of evidence. We now report the final data after 12 months of follow-up.

## Patients and methods

Patients meeting the following criteria were included into this trial: clinical diagnosis of OA of the knee and/or hand or finger joints, radiological proof of the diagnosis (plain radiographs), duration of anamnesis more than 3 months, and favorable general health status.

The joints were assigned to one of the following groups:

- Standard dose group: total dose of 3.0 Gy applied in single fractions of 0.5 Gy twice a week, and
- Experimental dose group: total dose of 0.3 Gy applied in single fractions of 0.05 Gy twice a week.

The dose applied was not known to the patients (single blinded).

Follow-up examinations were scheduled 3 months and 1 year after the end of radiotherapy and were performed by a physical examination of the patient in the hospital.

Primary endpoints were VAS (visual analogue scale) score, KOOS-PS [14] (knee injury and OA outcome score sum score—physical function short form), SF-SACRAH sum score [15] (short form score for the assessment and quantification of chronic rheumatic affections of the hands), and SF-12 [16] (short form 12, general health status) sum score. Secondary endpoints were SF-12 single scores and the use of analgesic medication.

The trial protocol was approved by the Ethikkommission der Ärztekammer des Saarlandes Saarbrücken (No. 60/17 on 19 April 2017). Furthermore, it was approved by the expert committee of the DEGRO (German Society for Radiation Oncology). The research was designed and carried out in agreement with the Declaration of Helsinki in its current version.

Further details of this trial protocol have been published in the first paper about this trial [1] and in the German Clinical Trials Register (DKRS00011870).

## Results

**Fig. 1:**
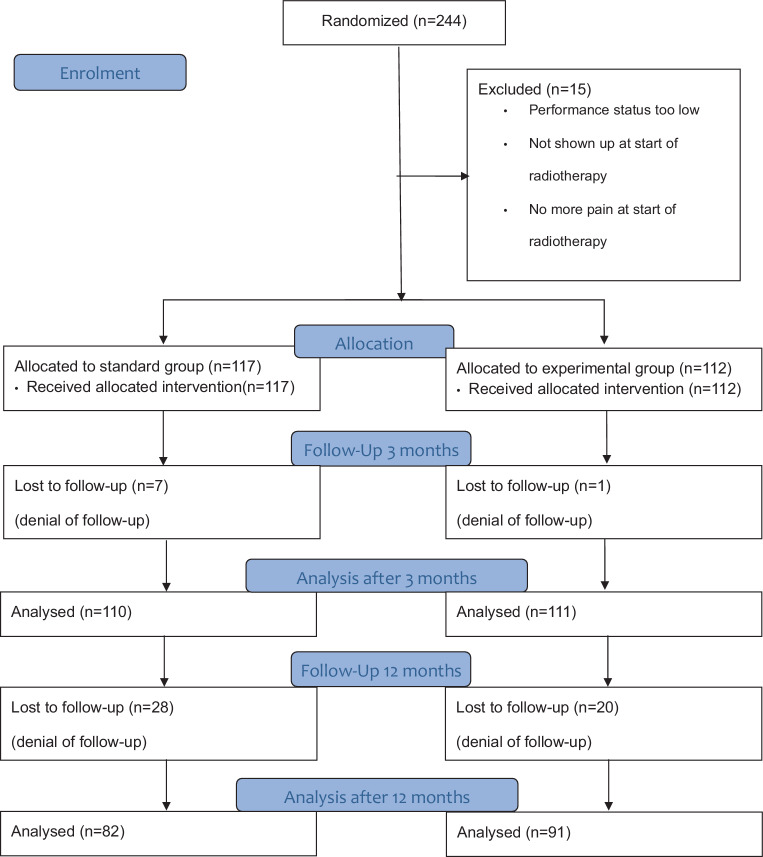
Consort diagram, updated

A total of 244 joints (in total 133 patients) were included in this trial. The majority of 220 joints were included at the Saarland University Medical Center in Homburg and 24 at the University Hospital of Regensburg. A total of 15 joints had to be excluded due to various reasons (poor health status, pain resolution at the planned start date of radiotherapy, did not shown up for radiotherapy; Fig. 1). Of the remaining 229 joints, 117 were randomized to the standard dose group and the remaining 112 to the experimental dose group. Of those, 110 joints in the standard dose group and 111 joints in the experimental dose group could be followed for at least 3 months. The mean follow-up of the cohort was 12.5 months (for further details see Fig. 1).

## Comparison of patient groups before radiotherapy

The mean age at enrolment was 68.2 years (median 67.9 years, interquartile range [IQR] 19) in the standard dose group and 66.3 years (median 64.8 years, IQR 16) in the experimental dose group (n. s.). The mean duration of pain anamnesis prior to the start of radiotherapy was 56.2 months (median 36 months, IQR 72, standard dose group) and 49.6 months (median 36 months, IQR 10, experimental dose group, n. s.). Furthermore, the groups were well balanced with regard to extension and onset of pain, impact of pain on daily life, daily work and leisure as well as previously applied treatments. There was a trend towards a higher percentage of hand joints in the experimental dose group (*p* = 0.559) and a significantly higher use of ice treatment in the standard dose group (*p* = 0.01) which was not regarded to be of clinical significance.

The VAS scores before radiotherapy were not significantly different between the groups (*p* = 0.209). In addition, the functional scores (KOOS-PS for the knee joints and SF-SACRAH for the hand and finger joints) were not significantly different (*p* = 0.527 and *p* = 0.551, respectively). As to the SF-12 scores, there was a trend in the somatic–doctor score and the somatic–patient score in favor of the experimental dose group (*p* = 0.058 and *p* = 0.060, respectively). Further details are depicted in the previous publication [1].

## Results after 12-month follow-up

**Fig. 2:**
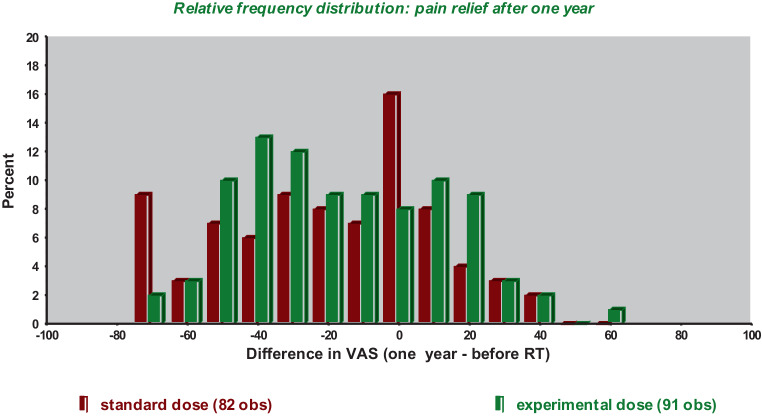
Detailed response to radiotherapy (*RT*) after 1 year. *VAS* visual analog score

In summary, we recorded a good analgesic effect of radiotherapy (difference of VAS scores 3 months after vs. those before radiotherapy) in both groups (results in the experimental group in parentheses; Fig. 2):

- Markedly improved (DeltaVAS ≥ 30 points): 41% (44%),
- Improved (0 < DeltaVAS < 30): 20% (21%),
- Stable 18% (8%), and
- Worse 21% (27%).

There were no statistically significant differences between the groups.

The mean difference in the VAS scores after 12 months compared to those before therapy was 19.5 in the standard dose group and 16.2 in the experimental dose group (*p* = 0.641). A similar result was achieved for the SF-SACRAH score (*p* = 0.663). Furthermore, we found no reasonable significance for the KOOS-PS score (*p* = 0.049) in favor of the standard dose group.

The majority of results concerning quality of life adequately matched those concerning pain and functional impairment. No statistically significant differences could be found in the following scales: physical score, doctor’s judgement: *p* = 0.492; physical score, patient’s judgement: *p* = 0.265; mental score, patient’s judgement: 0.226. There was a significant difference in the mental score, doctor’s judgement (*p* = 0.008) in favor of the standard dose group which was regarded clinically irrelevant. No acute side effects were recorded. Further details are depicted in Table 1.

**Table 1:** Comparison of pain/function/quality of life data 12 months after radiation therapy (RT) to those before RT

Item (difference of scores 3 months after RT−scores before RT)	Value	Standard dose group	Experimental dose group	*p*
VAS score	*n*	82	91	–
	Mean	−19.5	−16.2	–
	SD	30.3	28.3	–
	Minimum	−80	−70	–
	Maximum	40	60	–
	*p*	–	–	0.641
KOOS-PS score (knee joints)	*n*	23	20	–
	Mean	0.3	7.2	–
	SD	7.2	6.5	–
	Minimum	−18	−20	–
	Maximum	14	5	–
	*p*	–	–	0.049
SF-SACRAH score (hand joints)	*n*	58	69	–
	Mean	−7.5	−6.6	–
	SD	11.3	11.0	–
	Minimum	−38	−32	–
	Maximum	14	24	–
	p	–	–	0.973
SF-12 somatic doctor	*n*	44	51	–
	Mean	5.5	3.6	–
	SD	14.0	9.9	–
	Minimum	−28	−22	–
	Maximum	38	21	–
	*p*	–	–	0.492
SF-12 psychic doctor	*n*	44	51	–
	Mean	1.9	−1.3	–
	SD	7.3	10.6	–
	Minimum	−28	−30	–
	Maximum	15	26	–
	*p*	–	–	0.008
SF-12 somatic patient	*n*	44	51	–
	Mean	6.3	3.6	–
	SD	13.5	10.1	–
	Minimum	−26	−17	–
	Maximum	35	29	–
	*p*	–	–	0.255
SF-12 psychic patient	*n*	44	51	–
	Mean	0.5	−0.5	–
	SD	7.2	10.4	–
	Minimum	−31	−30	–
	Maximum	13	26	–
	*p*	–	–	0.226

*SD* standard deviation

*Visual analog score (VAS) scale* linear scale, 0 = no pain, 100 = maximum imaginable pain. Improvement = negative values

*KOOS-PS (knee joints)* 7 items, 0 = no functional impairment, 100 = maximum impairment; Improvement = negative values

*SF-SACRAH (hand joints)* 7 items, 0 = no functional impairment, 50 = maximum impairment, improvement = negative values

*SF-12 scales* 12 items, high values = favorable quality of life, Improvement = positive values

## Results in the time interval between 3- and 12-month follow-up

The mean difference in VAS scores after 12 months compared to those after 3 months was 0.55 in the standard group and 0.71 in the experimental group (*p* = 0.879) which was very small compared to the values after 3 months (−19.5 in the standard group and −15.8 in the experimental group).

The comparison of the KOOS-PS score resulted in a significant difference in favor of the experimental dose group (*p* = 0.008); in the SF-SACRAH scores there were no differences. In the SF-12 subscores no statistically significant differences were found (physical score, doctor’s judgement: *p* = 0.846; mental score, doctor’s judgement; *p* = 0.264; physical score, patient’s judgement: *p* = 0.537; mental score, patient’s judgement; *p* = 0.530). Further details are depicted in Table 2.

**Table 2:** Comparison of pain/function/quality of life data 12 months after radiation therapy (RT) to those 3 months after RT

Item (difference of scores 3 months after RT−scores before RT)	Value	Standard dose group	Experimental dose group	*p*
VAS score	*n*	82	92	–
	Mean	0.55	0.7	–
	SD	21.8	29.3	–
	Minimum	−70	−70	–
	Maximum	70	80	–
	*p*	–	–	0.879
KOOS-PS score (knee joints)	*n*	24	21	–
	Mean	2.6	−2.1	–
	SD	5.6	6.3	–
	Minimum	−5	−22	–
	Maximum	21	4	–
	*p*	–	–	0.008
SF-SACRAH score (hand joints)	*n*	57	70	–
	Mean	−1.8	−1.6	–
	SD	8.9	9.9	–
	Minimum	−23	−25	–
	Maximum	27	39	–
	*p*	–	–	0.938
SF-12 somatic doctor	*n*	44	51	–
	Mean	0.5	0.3	–
	SD	12.4	9.3	–
	Minimum	−30	−20	–
	Maximum	27	24	–
	*p*	–	–	0.846
SF-12 psychic doctor	*n*	44	51	–
	Mean	0.3	−1.8	–
	SD	8.6	10.1	–
	Minimum	−29	−34	–
	Maximum	18	30	–
	*p*	–	–	0.264
SF-12 somatic patient	*n*	44	51	–
	Mean	1.3	0.8	–
	SD	11.5	9.0	–
	Minimum	−27	−21	–
	Maximum	28	27	–
	*p*	–	–	0.537
SF-12 psychic patient	*n*	44	51	–
	Mean	0	−1.4	–
	SD	9.0	9.8	–
	Minimum	−29	−27	–
	Maximum	19	16	–
	*p*	–	–	0.530

*SD* standard deviation

*Visual analog scale (VAS) scale* linear scale, 0 = no pain, 100 = maximum imaginable pain. Improvement=negative values

*KOOS-PS (knee joints)* 7 items, 0 = no functional impairment, 100 = maximum impairment; Improvement = negative values

*SF-SACRAH (hand joints)* 7 items, 0 = no functional impairment, 50 = maximum impairment, improvement = negative values

*SF-12 scales* 12 items, high values = favorable quality of life, Improvement = positive values

## Prognostic factors

The only significant prognostic factor was the pain intensity before radiotherapy (univariate search, Spearman and Kendall *p* = 0.001) so that patients with a higher VAS score at the beginning of radiotherapy responded more favorably than those with lower VAS score. Age, location of pain, time from onset of pain and dose were not found to be significant prognostic factors as well as the Eaton score (hands) and the Kellgren score (knees). These results were confirmed by logistic regression analysis using the same variables mentioned above.

## Influence of the second radiotherapy course

As stated above, the patients with an insufficient response to the first radiotherapy course (*n* = 72) were offered a second one with the standard cumulative dose of 3.0 Gy. Of those, 35 patients had been randomized to the standard dose group, while 37 patients were members of the experimental dose group.

Expectedly, after 3 months we noticed a far better result for the patients with only one radiotherapy course (no need for second irradiation) compared to those with a need for a second course (data from the experimental group in parentheses):

- DeltaVAS one series: −24.5 (−24.4), difference not significant,
- DeltaVAS two series: −7.4 (−0.5); difference: *p* = 0.005 (*p* < 0.001).
  After 12 months, we found similar values:
- DeltaVAS one series: −26.8 (−22.4), difference, not significant,
- DeltaVAS two series: −8.9 (−4.8); difference *p* = 0.012 (*p* = 0.012).

Between 3 and 12 months after radiotherapy, there were only minimal changes, and significant differences were not found. Comparison of the functional scores and the quality of life data resulted in no statistically significant differences. Thus, we can assume that in nonresponders the effect of the experimental dose may be inferior compared to that of the standard dose. The second radiotherapy series was found to have no positive effect in nonresponders.

## Discussion

The aim of this study was to examine whether the analgesic effect of the standard dose is superior compared to that of a very low dose (taken as a kind of placebo dose). In summary, we found good pain relief in both groups, whereas there were no statistically significant differences between the dose groups. The improvement of pain mainly occurs during the first 3 months after radiotherapy. In the 9 months afterwards, we noticed only marginal additional improvement and pain relief—if achieved—persists over the long term.

Patients receiving a second radiotherapy course were classified as nonresponders. Apparently, their initial grade of pain relief was much lower than in the responders. The second radiotherapy course which was offered to those patients a minimum 3 months after primary radiotherapy had only a marginal effect.

The KOOS-PS values were significantly different in the time interval between 3 months and 1 year after radiotherapy. This is in agreement with the deterioration of KOOS-PS in this time interval. To our opinion, the functional symptoms—after an initial improvement—worsen over the long term.

In our collective, patients with a higher VAS score before radiotherapy had a better response than those with a lower intensity of pain.

Retrospective studies published in the past 80 years have shown favorable results concerning pain relief. We are well aware that these trials are of variable quality, the vast majority of the patients were treated using orthovoltage machines and doses of 6 Gy. The older results have been summarized in the S2k guideline of the DEGRO (German Society for Radiation Oncology) [2]. To our knowledge, there are only a few data available about small joints exclusively, which also showed good results [5, 6, 17]. More recent retrospective trials were published by Koc et al. [18], Hautmann et al. [19], Micke et al. [20], Rühle et al. [21], and Donaubauer et al. [22]. All of these authors state a significant response of pain to radiotherapy. Hautmann et al. published an additional paper about re-irradiation in patients with insufficient response to the first radiotherapy series or recurrent pain and regarded a second series as very effective [23]. A systematic review was written by Minten et al. [24]. They summarized that at that time (2016) insufficient data did not allow for a valid conclusion to be drawn on the efficacy of radiotherapy. The most recent review has been published by Dove et al. [25] who state that low-dose radiotherapy has an analgesic effect, while a lot of details are still unknown.

Three patterns-of-care studies have shown that low-dose radiotherapy for painful osteoarthritis is applied frequently in German-speaking countries [26–28].

Two very well designed randomized, controlled and double blinded trials were published in 2018 and 2019 (Minten et al. [29] and Mahler et al. [29]) showing no significant benefit for real radiotherapy as opposed to mock irradiation. These papers were published when patients were still being recruited for our trial.

We are well aware of the limitations of this trial. This trial had to be closed prematurely due to slow recruitment of patients. Furthermore, it appears plausible that single patients may have guessed their dosage arm especially when at least two joints in a patient were irradiated with different doses. The influence of oral medication during this trial was not assessed—to our opinion it was not ethical to limit intake of the oral analgesics.

It remains an issue of discussion why we found an equally negative result in agreement with the study of the Dutch colleagues irrespective of the fact that our trial was much larger. One reason may be the inhomogeneity in our patient collective. In discussions with the colleagues of the working group it was suggested to record the grade of the OA. Probably, in a relevant number of patients arthrosis was too advanced to allow a good radiation effect. An inflammatory and a degenerative pain component may be assumed. Thus. it may be a point of discussion whether only the inflammatory pain component responds to radiotherapy and not the degenerative one. This theory follows the fact that preclinical studies had an inflammatory arthritis model as a basis and no osteoarthritis model [7–9].

The optimal dose remains a point of debate. In the radiobiological literature, the highest effect was recorded after a dose of 0.5 Gy, whereas an effect on macrophages was found after 0.1 Gy [13]. Unfortunately, we could not find preclinical data for doses as low as 0.05 Gy in the literature. However, it will be interesting to examine such effects.

## Conclusions

Radiotherapy is efficient in yielding acceptable pain relief in the majority of patients with no observed adverse effects. However, we did not find a difference in the analgesic effect of a standard dose compared to a very low one. Explanations for these results are still lacking. Further trials comparing a standard radiotherapy dose to placebo and dose-finding studies applying different dose levels are recommendable. In our opinion, the indication to apply a second radiotherapy series in nonresponders can be discussed.
